# Action Direction of Muscle Synergies in Voluntary Multi-Directional Postural Control

**DOI:** 10.3389/fnhum.2017.00434

**Published:** 2017-08-30

**Authors:** Akari Kubo, Shota Hagio, Benio Kibushi, Toshio Moritani, Motoki Kouzaki

**Affiliations:** ^1^Laboratory of Neurophysiology, Graduate School of Human and Environmental Studies, Kyoto University Kyoto, Japan; ^2^Japan Society for the Promotion of Science Tokyo, Japan; ^3^Graduate School of Education, The University of Tokyo Tokyo, Japan; ^4^School of Health and Sport Sciences, Chukyo University Nagoya, Japan

**Keywords:** center of pressure, electromyogram-weighted averaging method, motor control, muscle synergy, non-negative matrix factorization

## Abstract

A muscle synergy is a coordinative structure of muscles that has been proposed as a strategy to reduce the number of variables that the central nervous system (CNS) has to address in motor tasks. In this article, the mechanical contribution of muscle synergies and coordinative structures of muscles in voluntary multi-directional postural control were investigated. The task for healthy, young subjects was to shift and align their center of pressure (COP) to targets dispersed in 12 different directions in the horizontal plane by leaning their bodies for 10 s. Electromyograms (EMGs) of 18 muscles and COPs were recorded in the experiment. Muscle synergies were extracted using non-negative matrix factorization (NMF), and the structure of coordinative modules to keep the posture leaning toward various directions was disclosed. Then the directional properties, such as the mechanical role (i.e., action directions, we use ADs as abbreviation below), of muscle synergies and muscles were estimated using an electromyogram-weighted averaging (EWA) method, which is based on a cross-correlation between the fluctuations in the activation of muscle synergies and the COP. The results revealed that the ADs of muscle synergies were almost uniformly distributed in the task space in most of the subjects, which indicates that mechanical characteristics reduce the redundancy in postural control. In terms of the composition of muscle synergies and the ADs of individual muscles, we confirmed that muscle synergies in multi-directional postural control comprised a combination of several muscles, including various ADs, that generate torque at different joints.

## Introduction

Humans maintain their bipedal standing posture by controlling their center of foot pressure (COP) to keep the projection of their center of mass on the ground within the base of support. In postural control during quiet standing, some studies have described human standing as a single inverted pendulum that pivots at the ankle joint (Winter et al., 1998; Peterka, 2002). However, recent studies have revealed that torque generated at joints other than the ankle (e.g., hip or knee) is negligible in explaining the mechanism for maintaining balance in reaction against perturbation (Kuo, 1995; Bloem et al., 2000; Alexandrov et al., 2005) and in a quiet stance (Aramaki et al., 2001; Creath et al., 2005; Hsu et al., 2007; Pinter et al., 2008). The majority of these studies were limited to postural control in the sagittal plane. However, studies have increasingly discussed postural control in the frontal plane or multiple directions (Carpenter et al., 1999; Grüneberg et al., 2005; Bingham et al., 2011). Torres-Oviedo and Ting (2007) showed that people use muscle synergies that individually correspond with ankle, hip and knee strategies in response to multi-directional support-surface transitions and that flexible reactions can be generated from a combination of these motor modules. Our recent study (Imagawa et al., 2013) revealed that several muscles that have different action directions (ADs), which are the mechanical characteristics of each muscle that represents endpoint force vectors (Kutch et al., 2010; Hagio and Kouzaki, 2015b), are co-activated in the multi-directional and intentional movement of the COP. This study indicated that lower leg muscles have ADs in either anterior-posterior or diagonal directions, such that COP movement in the right-left direction must be accomplished by cooperation among multiple muscles. This notion was proven by the index η, which reflects the degree of synergistic co-activation (Kutch et al., 2008). Therefore, humans co-activate several muscles that contribute to different strategies (i.e., ankle, hip and knee strategies) and have various ADs distributed in a limited range to accomplish multi-directional postural control.

This postural task involves multiple muscles around several joints; because it is redundant in musculoskeletal control, the central nervous system (CNS) must address a huge amount of information if it needs to send neural orders to these muscles. For this reason, complex and flexible motor control is considered to be impossible (Bernstein, 1967). To solve this problem, previous studies have proposed the necessity of modular organization, i.e., muscle synergies, to simplify the redundancy (Lee, 1984; Macpherson, 1991; Tresch et al., 1999; Hagio and Kouzaki, 2014, 2015a). Muscle synergies are low-dimensional structural units that are composed of several muscles. As per this theory, variables that the CNS should handle are reduced by controlling muscle synergies but do not independently address each muscle. Thus, humans can accomplish the desired task. Muscle synergies have been reported in various motor tasks: walking (Hagio et al., 2015), running (Nishida et al., 2017), pedaling (Raasch and Zajac, 1999) and reaching (d’Avella et al., 2006). Muscle synergies in postural control have also been discussed in numerous studies (Krishnamoorthy et al., 2003; Torres-Oviedo and Ting, 2007; Chvatal et al., 2011). The majority of these studies focused on the automatic postural responses or rapid and automatic postural reaction against perturbation but not voluntary control. The mechanical contribution of muscle synergies in intentional and multi-directional postural control (which are some of the most basic motor tasks) must be quantified because they may reflect the neural organization of the motor system. In addition, muscle synergies in postural control must be investigated in relation to COP fluctuations along a time sequence, which reflects the variability of the moment around the joints involved in standing.

These findings prompted the hypothesis that the ADs of muscle synergies recruited in voluntary multi-directional postural control would be evenly distributed in the task space to efficiently reduce the redundancy in the motor task. To examine the relationship between motor output and muscle synergies, the electromyogram-weighted averaging (EWA) method has been utilized in previous studies (Kutch et al., 2010; Imagawa et al., 2013; Hagio and Kouzaki, 2015b). The EWA method can extract the mechanical AD of each muscle or muscle synergy from an endpoint force based on the cross-correlation.

The goal of this study was to show the coordinative structure of muscles that contribute to voluntary postural control. The AD of each muscle synergy and/or muscle was estimated using the EWA method based on a cross-correlation between the fluctuations in the activation of muscle synergies (or electromyogram (EMG) traces of muscles) and COP. Then, the ADs of muscles and muscle synergies were compared; the results confirmed that the ADs of muscle synergies were distributed in a well-balanced and compensated manner that cannot be covered by a single muscle.

## Materials and Methods

### Subjects

Nine young healthy male subjects volunteered for this experiment. Their mean (±SD) age, height and body mass were 24.0 (±1.3) years, 174.6 (±4.3) cm and 68.4 (±6.3) kg, respectively. None of the subjects had a history of any neurological disorder and their vision had been corrected to normal levels. All subjects provided written informed consent to participate in the study after receiving a detailed explanation of the purposes, potential benefits and risks associated with participation. The experimental procedures used in this study were performed in accordance with the Declaration of Helsinki and were approved by the Committee for Human Experimentation at the Graduate School of Human and Environmental Studies at Kyoto University (Approval number 28-H-22).

### Experimental Protocol

The basic procedure for the setup and measurement of postural sway during multi-directional postural control has been described in our previous study (Imagawa et al., 2013) and is summarized in this study. The subjects stood barefoot on a force platform with their arms comfortably by their sides with minimal distance between their feet. During multi-directional postural control, the COP position was calculated in real time based on the vertical components of the force platform data and displayed on a monitor in front of the subjects for visual feedback. The original position of the COP was determined by the COP position at which the subject naturally performed idle standing for approximately 20 s. In each trial, subjects were instructed to move their COP from the original position to the target point by leaning their body around the ankle joint and hold the target COP as precisely as possible for approximately 10 s. After each trial, the subjects returned their COP to the original position and a subsequent trial began. The original position and the desired target in each trial were always displayed on a feedback monitor throughout the task. The duration of each trial was recorded when the COP was within a 0.3 mm radius from the desired target. The timer that showed the duration was only visible to the experimenter; thus, the subjects moved their COPs in accordance with the instructions (“Go” and “Return”) given by the experimenter. All subjects successfully maintained their COPs for 10 s at the desired position. The subjects were also guided to minimize their movement of knee and hip joints in shifting or maintaining their COP positions, so that they primarily employed an ankle strategy. As a result, any critical movement of knee or hip joints were not confirmed from the kinematic data. Twelve different target positions were placed at intervals of 30° and were located 30 mm from the original position. The experiment consisted of five blocks; in each block, the subjects were required to perform COP displacement toward 12 different targets that were presented in a randomized order. A sufficient rest period was allowed between blocks to prevent fatigue. Surface EMG data from the following 18 muscles that spanned ankle, knee and hip joints on the right side were recorded: tibialis anterior (TA), soleus (SOL), medial gastrocnemius (MG), lateral gastrocnemius (LG), fibularis longus (FL), rectus femoris (RF), vastus lateralis (VL), biceps femoris long head (BFl), biceps femoris short head (BFs), semimembranosus (SM), semitendinosus (ST), gluteus medius (Gmed), adductor longus (AL), sartorius (Sar), rectus abdominis (RA), erector spinae (ES), flexor digitorum longus (FDL) and extensor digitorum longus (EDL). To observe detailed activation of the muscles related to ankle strategy, data were recorded from the medial and lateral side of the SOL. The surface EMGs of the RF, VL, BFl, BFs, SM, ST, Gmed, AL, Sar, RA and ES were recorded to observe the cooperation between hip joint muscles or knee joint muscles for postural control, especially in the medio-lateral direction (Winter et al., 1996; Imagawa et al., 2013). To confirm that the subjects did not critically flex their hips or knees during the motor task, the kinematic data were also bilaterally measured at 100 Hz with the three-dimensional optical motion capture system. Infrared reflective markers were attached to each side of the subjects’ skin that overlaid the following body landmarks: temple, acromion, lateral condyle of the elbow, styloid process of the ulna, anterior superior iliac spine, posterior superior iliac spine, greater trochanter, lateral condyle of the knee, lateral malleolus, second metatarsal head and heel. The markers were also attached to the vertex, chin and right blade bones.

### Apparatus

The COP position calculated from vertical components of the ground reaction force and EMGs were recorded during the task. The ground reaction force was measured by a force platform (EFP-S-1.5kNSA13B, Kyowa, Tokyo, Japan). The EMGs were recorded using Ag-AgCl electrodes with a diameter of 5 mm and an inter-electrode distance of 10 mm. To prevent cross-talk among neighboring muscles, a small inter-electrode distance was employed (Hagio and Kouzaki, 2014, 2015a,b) to carefully obtain the site of electrode placement in each muscle with a B-mode ultrasonic apparatus (α-6, Aloka, Tokyo, Japan; Hagio and Kouzaki, 2015a). A reference electrode was placed on the right internal malleolus and right external malleolus. The EMG signals were amplified (MEG-6116M, Nihon-kohden, Tokyo, Japan) with bandpass filtering between 5 Hz and 1000 Hz. All electrical signals were stored with a sampling frequency of 2000 Hz on the hard disk of a personal computer using a 16-bit analog-to-digital converter (PowerLab/16SP; AD Instruments, Sydney, NSW, Australia). The customized software based on the LabView-15 package (National Instruments, Austin, TX, USA) was used to measure the duration of in each trial and display the feedback of the COP, the original position and the target position.

### Data Processing

For all recorded signals, data for a 5-s period were selected (the first and last 2.5 s were removed) for analysis of individual trials in which the COP trajectory was relatively constant. The COP data were filtered with a zero-phase-lag, fourth-order Butterworth bandpass filter from 5 Hz to 30 Hz to remove the effect of postural sway that is induced by voluntary contribution and non-physiological noise (Kutch et al., 2008). Raw EMG data were filtered with a zero-phase-lag, fourth-order Butterworth high-pass filter at 20 Hz to reduce the baseline noise and movement artifacts (De Luca et al., 2010), after which they were demeaned, rectified and low-pass filtered at 40 Hz (Chvatal et al., 2011). The EMGs and COP datasets were resampled into 100 points; however, for EWA analysis, the filtered traces were resampled into 1000 points. Datasets of five blocks for each target angle were pooled and analyzed.

### Extraction of Muscle Synergies

Non-negative matrix factorization (NMF) was employed to extract muscle synergies from the data matrix of the EMGs (M) collected from each subject (Lee and Seung, 1999; Tresch et al., 1999). The reconstruction of the data is conducted as the following equation:

$$\text{M}=\sum_{i=1}^{N}W_{i}C_{i}+\;\varepsilon \;(W_{i}\geq 0,C_{i}\geq 0)\;$$

where *W*_i_ indicates the ratio of the contribution of each muscle to the synergy *i*. The composition of the muscle synergies was constant across trials; however, the activation coefficient *C*_i_ that represents the magnitude of activation of the synergy *i* in each direction can change. The column of *C*_i_ consisted of 30,000 variables (5 blocks × 12 directions × 5 s × 100 samples). *ɛ* is the residual of the reconstruction. The synergy weighting and activation coefficient matrices were normalized with the norm of muscle-weighting vector such that the individual muscle-weighting vector was a unit vector. To determine the number of muscle synergies *N*, cross-validation was employed. First, EMG data were shuffled across muscles and muscle synergies were extracted from 80% of the data. Second, the remaining 20% of the data was reconstructed by the muscle synergies obtained by the first procedure. The mean and SD of the variability accounted for (VAF) were obtained by repeating this operation 30 times, and the number of muscle synergies *N* was chosen based on the global VAF and muscle VAF. The former denotes the accuracy of the reconstruction of the whole EMGs (M), and the latter denotes the reconstruction of the EMG of each muscle. *N* was determined such that the global VAF exceeds 90% and the mean muscle VAF exceeds 80%. VAF was defined as 100× the coefficient of determination from the uncentered Pearson’s correlation coefficient (Torres-Oviedo et al., 2006). The equation for VAF computation is expressed as:

$$VAFglobal=1-\frac{\sum_{i=1}^{I}\sum_{j=1}^{J}{\left(x_{ij}-{\hat{x}}_{ij}\right)}^{2}}{\sum_{i=1}^{I}\sum_{j=1}^{J}{\left(x_{ij}\right)}^{2}}$$

$$VAFmuscle_{i}=1-\frac{\sum_{j=1}^{J}{\left(x_{ij}-{\hat{x}}_{ij}\right)}^{2}}{\sum_{j=1}^{J}{\left(x_{ij}\right)}^{2}}$$

where *x*_ij_ is a sample of the original EMG data of muscle *i* at time *j*, whereas ${\hat{x}}_{ij}$ is a sample of the reconstructed data produced by NMF; *I* and *J* denote the number of muscles (19 muscles) and the number of samples along a time series (5 blocks × 12 directions × 5 s × 100 samples), respectively.

The similarity between any two synergies was measured by normalizing the synergy vectors (Euclidean norm) and computing their scalar product (*r* > 0.58; *p* < 0.01; Gentner et al., 2013). If two synergies in one subject were assigned to the same synergy group, a pair of synergies with the highest correlation was defined as the same group of synergies.

### EWA Analysis

**Figure 1:**
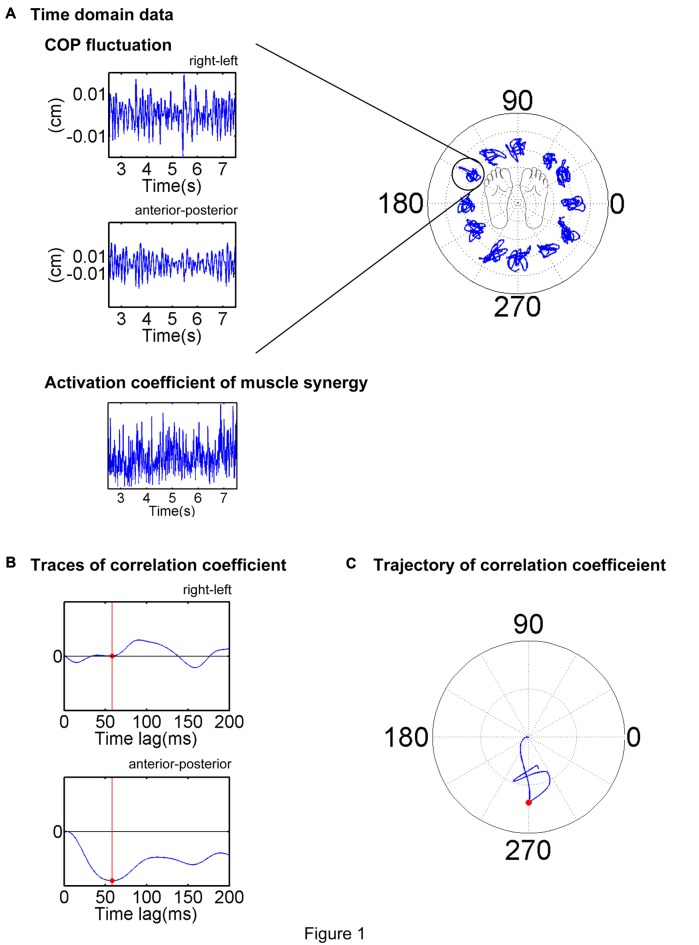
Description of the electromyogram-weighted averaging (EWA) method. EWA analysis of a representative trial is illustrated. **(A)** Center of pressure (COP) and activation coefficient traces in the stable period (middle 5 s or 2.5–7.5 s of the trial) were extracted and analyzed. **(B)** Cross-correlation between the fluctuations in the COP and activation coefficient was calculated for each time lag of 0–200 ms. **(C)** EWA direction was determined as the first peak magnitude of the trajectory of the correlation coefficient, as indicated by a red dot.

To determine the direction in which each muscle and muscle synergy were recruited to move the COP, the EWA method was employed (Kutch et al., 2010; Imagawa et al., 2013). This method is based on a cross-correlation between the fluctuations in the activation coefficients of the muscle synergies and COP (Figure 1A). For the EWA analysis, the activation coefficients were calculated for each 1000 samples per 1 s. This analysis was performed over an approximately steady period of COP in left-right and anterior-posterior directions of COP fluctuation for 5 s of the time course in the prior analysis. Surface EMG recordings from a muscle *EMG*_m_(*t*) (*t* denotes a discrete time point) or activation of a synergy *C*_s_(*t*); two COP components (*COP*_L-R_: left–right direction, *COP*_A-P_: anterior–posterior direction) were employed for cross-correlation. The EWA trajectories were calculated using the following equations:

$$EWA_{mL-R}(i)=\sum_{t}COP_{L-R}(i+t)EMG_{m}(t)\;$$

$$EWA_{mA-P}(i)=\;\sum_{t}COP_{A-P}(i+t)EMG_{m}(t)$$

$$EWA_{sL-R}(i)=\;\sum_{t}COP_{L-R}(i+t)C_{s}(t)\;$$

$$EWA_{sA-P}(i)=\;\sum_{t}COP_{A-P}(i+t)C_{s}(t)$$

where summation was performed over the extracted time intervals. The EWA trajectory in the horizontal plane was shifted to enable it to start from the origin at the zero time lag. Each EWA was temporally quantified and spatially based on a time lag from 0 ms to 200 ms, during which the EWA trajectory attained its first peak magnitude. This time lag was used to define the EWA time-to-peak; the corresponding spatial direction was used to define the EWA direction of each trial (Figures 1B,C). The EWA direction was selected on the condition that the correlation coefficient was statistically significant (*p* < 0.05), and the time-to-peak value ranged between 50 ms and 150 ms, that is, the COP fluctuations were reasonably attributed to activation of the muscle or muscle synergy considering electromechanical delay (Vos et al., 1990; Hagio and Kouzaki, 2015b). Then the ADs of each muscle and muscle synergy were defined by averaging the EWA vectors of the chosen trials. The CircStat toolbox of MATLAB (Berens, 2009) was employed for this calculation.

To confirm the validity of the analysis in determining ADs, methodological identifications was conducted; the ADs determined by the EWA method reflected the physiological characteristics of muscles or muscle synergies but did not reflect secondary products of the methodology. Time elements of EMG data or activation traces of muscle synergies were shuffled and conducted the EWA analysis. By repeating this procedure 100 times, 95% bootstrap confidence intervals of ADs estimated from randomized data were gained.

## Results

### Muscle Synergy

**Figure 2:**
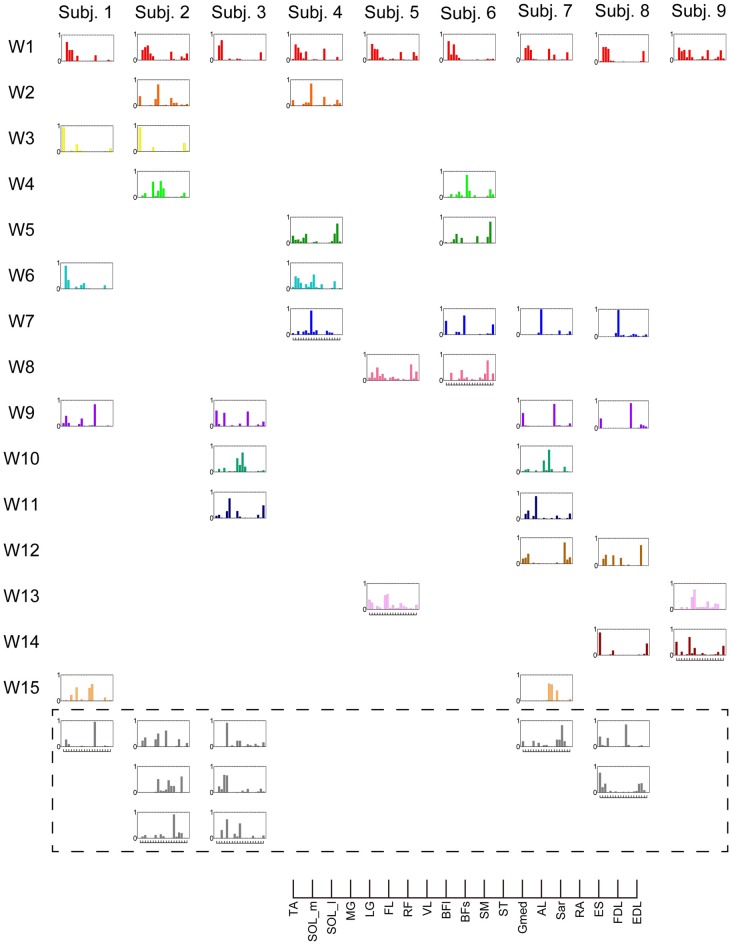
Muscle synergies in subjects. Extracted muscle synergies were grouped using cosine similarity. The color of the synergies in the same group are identical. Subject-specific muscle synergies are indicated by a gray color.

**Figure 3:**
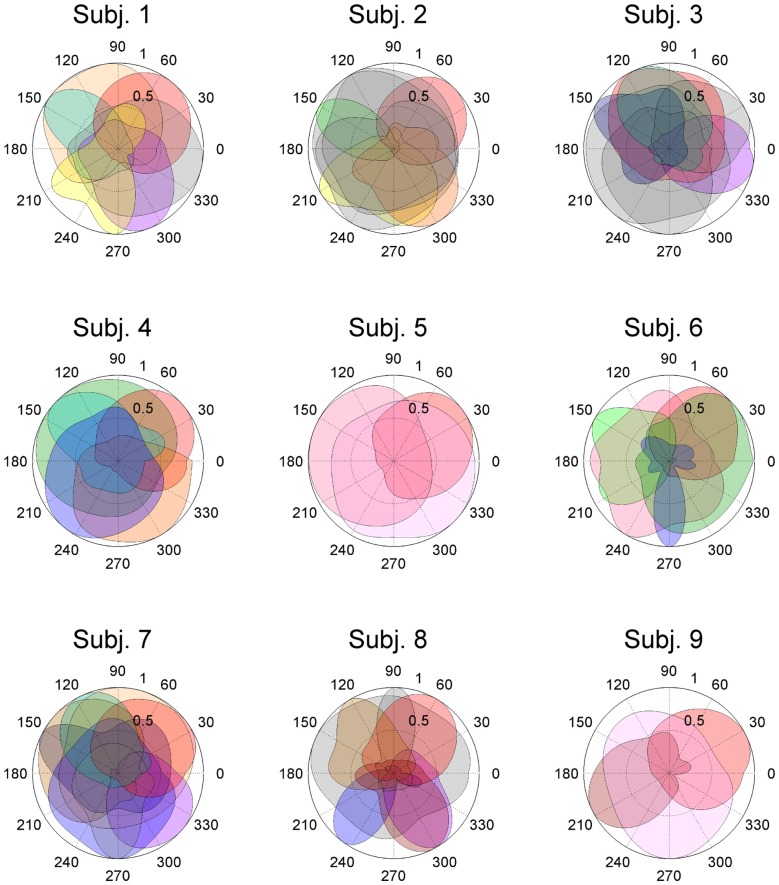
Activation coefficients of muscle synergies in subjects. Activation coefficients of each muscle synergy were averaged for each trial, normalized by the maximum magnitude, and polar plotted. Colors correspond to Figure 2. The numbers at the peripheries of the circles (0.5 and 1) denote the magnitude of the activation coefficients of the muscle synergies. They were normalized by the maximum activation.

In this study, entire muscle activities were accounted for by 5.7 ± 1.80 (mean ± SD) global muscle synergies, which were extracted from the entire data set of five blocks across each subject. Figures 2, 3 show the global muscle synergies and the associated activation coefficients in the five blocks for all the subjects. The polar plot (Figure 3) represents the averaged activation coefficients of the muscle synergies in different target directions for each subject. The magnitude of the averaged activation coefficients of each synergy was normalized by the maximum activation coefficient of the muscle synergy. Twelve averaged activation coefficients across the 12 directions were interpolated into 360 points.

The synergy W1 was primarily comprised of triceps surae muscles (SOL, MG and LG) and Gmed. It was activated when the subjects maintained the COP in the right anterior of the horizontal plane, and was similar among all subjects. The synergy W2 included TA, VL and Gmed. It was activated in maintaining the COP in the right posterior and was similar among Subject 2 and 3. The synergy W3, which was applied when the COP was held in the left posterior direction, comprised of TA and FL and was similar in Subject 1 and 2. The synergy W4 primarily mobilized FL, BFl, BFs and EDL, and was similar in Subject 2 and 6. It was activated when the COP was kept left anterior. The synergy W5 was dominated by the activation of ES and EDL, and was recruited in keeping the COP at relatively broad directions. It was similar in Subject 4 and 6. The synergy W6 consisted of BFl, BFs, ES and SOL, and was employed when the COP was kept left anterior. It was similar in Subject 1 and 4. The synergy W7, which included SOL, EDL and ES, was activated in maintaining COP leftward and was similar among Subject 4, 6, 7 and 8. The synergy W8 primarily activated VL and was recruited when the COP was kept posterior. It was similar in Subject 5 and 6. The synergy W9 consisted of the lower leg muscles (TA, SOL, MG and FDL) and Gmed. It was activated in maintaining the COP rightward; this result was similar in Subject 1, 3, 7 and 8. The synergy W10 recruited BFl and SM, and was activated in maintaining COP at left anterior. It was similar between Subject 3 and 7. The synergy W11 included FL and EDL. It was mainly activated in maintaining the COP in the left anterior and was similar among Subject 3 and 7. The synergy W12, which was applied when the COP was held in left anterior, was constructed by SOL and ES and was similar in Subject 7 and 8. The synergy W13 primarily mobilized RF and VL, and was similar in Subject 5 and 9. It was activated in broad conditions. The synergy W14 was dominated by the activation of TA, FL and EDL, and was recruited in keeping the COP at posterior. It was similar in Subject 8 and 9. The synergy W15 consisted of SM and ST, and was employed when the COP was kept anterior. It was similar in Subject 1 and 7. The remainder of the muscle synergies were subject-specific (Figure 2, gray bars). As a result, the composition of the muscle synergies revealed that the majority of the extracted muscle synergies were related to multi-segment movement by recruiting muscles of the trunk or thigh with lower leg muscles or using bi-articular muscles, even if the subjects were instructed to primarily apply the ankle strategy. However, some subjects had synergies that correspond to movement around a single joint. W3 primarily included mono-articular muscles in the lower leg, which contributed to the generation of torque around the ankle joint. W8 recruited VL, which is a mono-articular muscle involved with the production of torque at the knee joint. In addition, it seemed that the majority of the extracted muscle synergies exhibited their maximum activation in a diagonal direction.

### Action Direction

**Figure 4:**
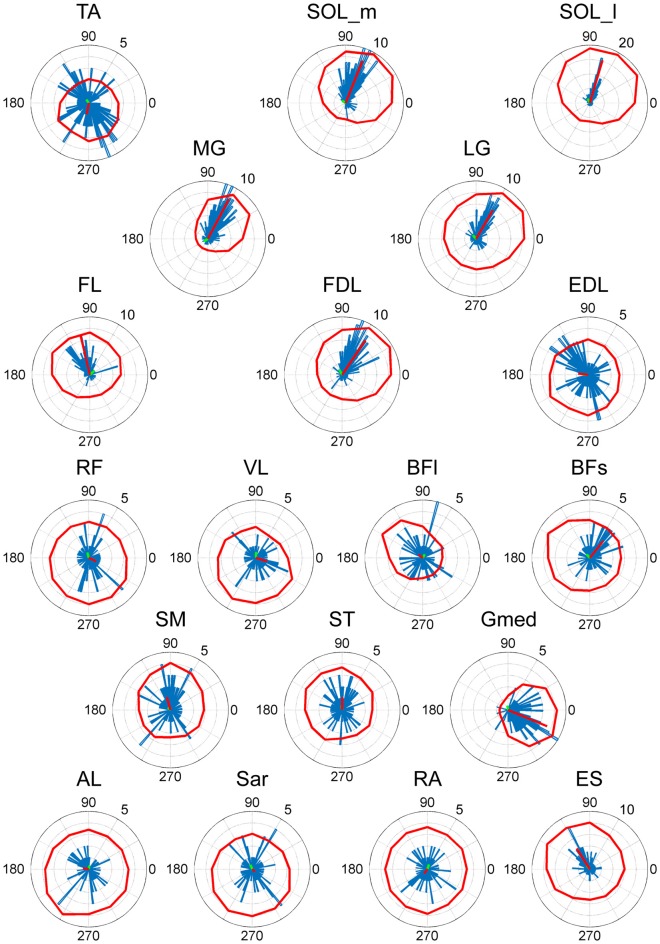
EWA direction of muscle activities. Valid EWA directions of each muscle are displayed as blue histograms. Red polar plots show the averaged electromyogram (EMG) data. EMG polar plots in each figure are magnified for visibility. The numbers at the peripheries of the circles represent the frequency of recruitment of the muscle associated with the direction.

**Figure 5:**
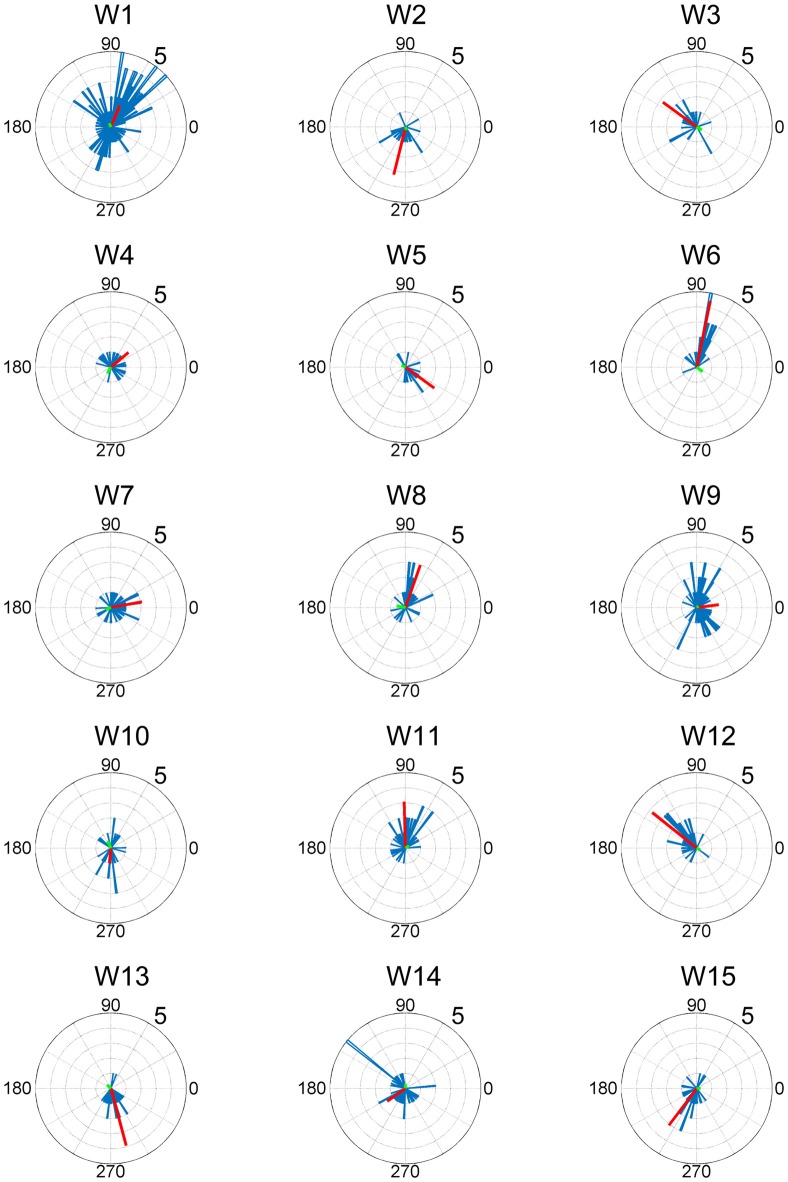
EWA direction and action direction (AD) of muscle synergies. The AD was calculated based on the EWA directions using circular statistics. The red lines represent the estimated ADs of each muscle synergy. The numbers at the peripheries of the circles denote the frequency of recruitment of the muscle synergy associated with the direction.

As a result of EWA, all muscles and muscle synergies exhibited statistically significant ADs (Figures 4, 5), which refers to the shuffled data. The analysis correctly extracted the physiological characteristics of muscles or muscle synergies. ADs of the lower leg muscles were distributed between 60° and 120° in the horizontal plane, with the exception of TA (Figure 4). Muscles in the thigh or trunk were engaged in relatively various directions, including anterior-posterior, right-left and diagonal directions. Lower leg muscles, especially the triceps surae muscles FL and Gmed indicated a remarkably strong tendency of ADs.

**Figure 6:**
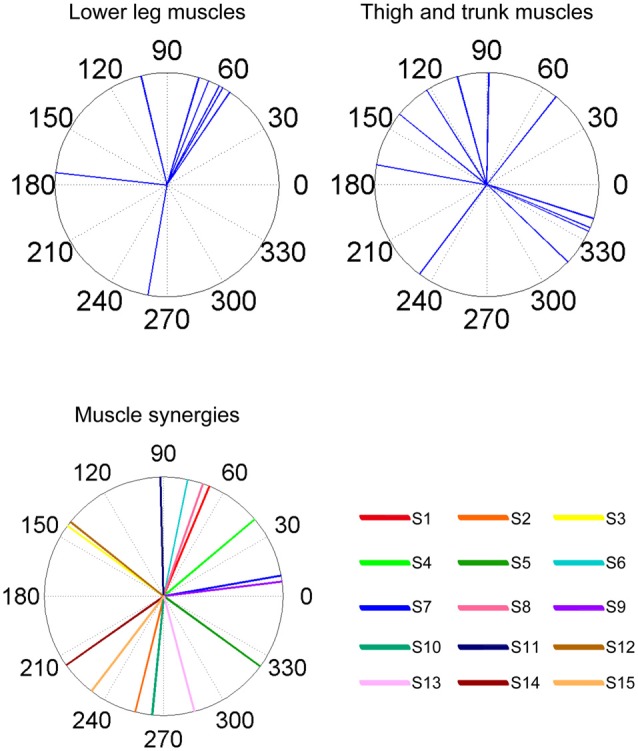
Summary of ADs of muscles and muscle synergies. The upper panels indicate the ADs of the lower leg muscles (left) and the thigh and trunk muscles (right). The lower panel indicates the ADs of each muscle synergy. The AD was calculated based on the EWA directions using circular statistics.

**Figure 7:**
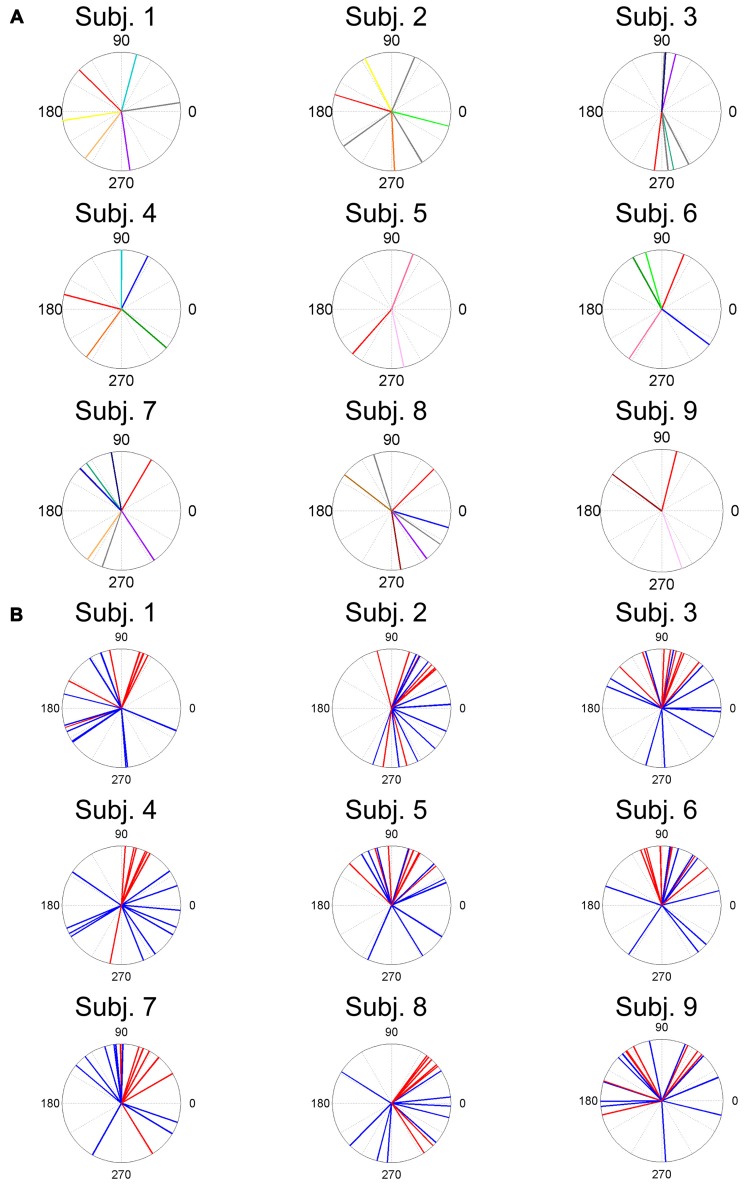
ADs of muscle synergies **(A)** and related muscles **(B)** for each subject. The different color lines indicate different muscle synergies **(A)**. Red lines and blue lines indicate lower leg muscles and thigh and trunk muscles, respectively.

The ADs of muscle synergies were distributed in various directions in most of the subjects (Figures 6, 7). This distribution seems to be well-balanced considering that the ADs of individual muscles, especially the lower leg muscles, were the main working muscles involved in the posture task.

## Discussion

The objective of this research was to analyze the coordinating structures of muscles that contribute to voluntary postural control. Using NMF from the EMG dataset, 5.7 ± 1.80 muscle synergies were extracted and sorted according to cosine similarity. The ADs of the muscle synergies were estimated using the EWA method (Kutch et al., 2010; Hagio and Kouzaki, 2015b), which is based on cross-correlation between an activation coefficient and the COP. The results confirmed that their ADs were distributed in well-balanced directions, which proves that muscles synergies contribute to the simplification of postural control by reducing the redundancy caused by a large number of muscles involved in bipedal standing.

### Muscle Synergy Contribution in Voluntary Postural Control

ADs of lower leg muscles tend to be distributed toward the anterior-posterior direction, as indicated by our previous study (Imagawa et al., 2013). Thigh and trunk muscles had more dispersed ADs, including the right-left direction (Figure 4). The combinations of these muscles enable the control of posture in multiple directions by producing the required direction of force. However, the combinations of muscles and the magnitudes of these activations will exhibit a distinct pattern if these muscles were individually controlled, which creates redundancy and hinders subtle postural control (Bernstein, 1967). During the postural maintenance employed in this study, muscles around the ankle joint primarily contributed to postural control. The results indicate that the ADs of muscle synergies were almost uniformly distributed in the horizontal plane (Figures 5, 6). This finding indicates not only a decrease in the number of targets to which the CNS has to send commands but also a reduction in the patterns of activation and combination of modules from which the CNS must choose. Recruiting these muscle synergies but not individual muscles produces a force vector of various directions in a simple manner and contributes to a reduction in the redundancy in postural control. In addition, some muscle synergies had ADs that substantially differed from any ADs of composing muscles, which indicates that muscle synergies not only simplify the redundancy of the musculoskeletal system but also generate a force vector that a single muscle cannot bear. This finding also exhibited the activation pattern (Figure 3). The maximum activation of most of the extracted muscle synergies occurs in a diagonal direction. Because the mechanical directions of lower leg muscles were primarily distributed in the sagittal plane in our experiment, diagonal force must be produced by the cooperation of several muscles, including the trunk or thigh (Imagawa et al., 2013).

### Similarity of Muscle Synergies Across Subjects

The results indicate that a few muscle synergies were common among subjects; the synergy W1 was the only synergy that was similar among all subjects (Figure 2). It transferred the COP to the left posterior when subjects kept the COP to the right anterior (Figure 3). The remaining synergies were similar in a maximum of two subjects, which indicate that subjects used their combinations of muscles to produce a multi-directional force vector. As shown in Figure 7A, even similar muscle synergies among subjects indicated different ADs among subjects. These results are attributed to several reasons: the subtle difference in the weight of each muscle in muscle synergies (Figure 2) or the difference in the AD of the same muscles among subjects (Figure 7B). This difference may be attributed to habitual factors, such as slight differences in posture or innate factors such as musculoskeletal configuration. Hagio and Kouzaki (2015b) indicated that the AD of a single muscle synergy may be varied due to the methodology of EWA. The production of cross-correlations between activation traces of target muscle synergies and the COP reflects synchronous force generation of several muscle synergies. This research noted that the variability in the AD of a muscle synergy arises because the direction of force produced by a muscle synergy reflects a pulling direction of recruited motor units. Regarding the hypothesis that the neural basis of muscle synergies lies in spinal interneurons (Hart and Giszter, 2010; Overduin et al., 2014), the mechanical feature of a muscle synergy can vary depending on which motor units to mobilize because they have a broad range of pulling directions (Thomas et al., 1986, 1990). Despite the variability of the ADs among subjects, the ADs of muscle synergies within each subject balanced well in various directions (Figure 7A). Each subject has an individual method for reducing redundancy in postural control.

### Action Direction and Activated Direction of Muscle Synergies

Figure 6 shows that the direction in which the muscle synergy activated was not always the same as the AD of the muscle synergy because activation indicates the temporal function of the muscle synergy (in which task the muscle synergy activated to shift the COP), while the AD shows the spatial function of the muscle synergy (where the COP moved by activating the muscle synergy). Because the experiment conducted in this study was maintaining posture, these two factors did not always apply in the same direction.

### Strategies for Postural Control in Multiple-Directions

The majority of the extracted muscle synergies were composed of both lower leg muscles and thigh or trunk muscles and were related to the motion of the ankle, hip and knee joints. This finding indicates that voluntary control of the COP cannot be accomplished by the ankle even if angle changes in knee and hip joints are vanishingly small as compared to ankle joint angle changes; therefore, a mixture of several strategies is essential because numerous studies have revealed (Kuo, 1995; Aramaki et al., 2001; Alexandrov et al., 2005; Krishnamoorthy et al., 2005; Hsu et al., 2007; Pinter et al., 2008). Previous studies have indicated that a hip strategy can change the hip joint angle faster than an ankle strategy modifies the ankle joint angle due to a larger amplitude (Runge et al., 1999; Bloem et al., 2000). The mixture of these different features of various strategies is important for precise and flexible postural control (Creath et al., 2005).

## Conclusions

Voluntary and multi-directional postural control is one of the most basic motor tasks for humans; however, the manner in which muscles coordinate to bear force in various directions remains ambiguous. This study approached this question in terms of muscle synergies and their mechanical contribution in a multi-directional COP shift. The results indicated that ADs of muscle synergies were evenly distributed within the horizontal plane and that the reduction of redundancy in the musculoskeletal system works by combining muscles with different ADs and applying various strategies.

## Author Contributions

AK, SH, TM and MK: conception and design of the experiments; AK, SH and BK: collection, analysis and interpretation of data; AK, SH and MK: drafts of the article or critical revisions for important intellectual content; AK, SH, BK, TM and MK: final approval of the version to be published.

## Conflict of Interest Statement

The authors declare that the research was conducted in the absence of any commercial or financial relationships that could be construed as a potential conflict of interest.
